# Assessment of the validity error flags generated by the Technicon SMAC system and the Perkin-Elmer KA 150 enzyme analyser

**DOI:** 10.1155/S1463924682000200

**Published:** 1982

**Authors:** E. Arthur Robertson, André C. van Steirteghem, Donald S. Young

**Affiliations:** 1 Clinical Chemistry Service and Laboratory Computer Service Clinical Center National Institutes of Health Bethesda Maryland 20205 USA; 2 RIA Laboratorium Academisch Ziekenhuis Vrije Universiteit Brussel Laarbeeklaan 101 Brussels B 1090 Belgium; 3 Department of Laboratory Medicine Mayo Clinic Rochester Minnesota 55901 USA


Journal of Automatic Chemistry, Volume 4, Number 1 (April–June 1982), pages 72–74

### Assessment of the validity of error flags generated by the Technicon SMAC system and the Perkin-Elmer KA 150 enzyme analyser

**E. Arthur Robertson, André C. van Steirteghem and Donald S. Young**

*Clinical Chemistry Service and Laboratory Computer Service, Clinical Center, National Institutes of Health, Bethesda, Maryland 20205, USA*

### Introduction

The Technicon SMAC system and the Perkin-Elmer KA 150 kinetic enzyme analyser both incorporate electronic logic to monitor the progress of the analytic process. When atypical conditions are detected during an analysis, the result is flagged for reanalysis (in some cases it is simply not printed). An error flag indicates that noise, an inadequate plateau, an altered dwell time or some other problem has occurred, casting doubt on the accuracy of the final result. If the criteria for flagging results were too strict, many accurate results would be inappropriately flagged. Conversely, the use of lenient criteria would result in erroneous results not receiving an error flag.

The SMAC is a 20-channel continuous-flow biochemical analyser. Rather than plotting the individual peaks for each channel on a chart recorder, the SMAC uses an internal computer to monitor the peaks and perform the required calculations. Each peak is sampled four times/s. The computer compares each peak to an empirically derived 'ideal' peak for that channel. Checks are made to detect excessive noise, an unsatisfactory steady state, insufficient peak height, and premature or inadequate wash-out. If the peak does not pass these tests, an error flag is printed. After four consecutive unsatisfactory curves on the same channel, the computer will stop printing results for that channel.

The KA 150 enzyme analyser monitors the course of enzymatic measurements for indications of unsatisfactory analytical conditions. When a problem is detected, an error flag is printed along with the result. 'AC' signifies a nonlinear curve with upward concavity, 'DC' signifies a nonlinear curve with downward concavity, and 'HI' indicates excessive absorbance.

To evaluate the validity of the error flags printed by the SMAC and the KA 150 enzyme analyser, the authors made repeat analyses of multiple specimens obtained from the same individuals over a six-week period. Then, using each volunteer as his own control, the flagged results were compared with the unflagged results for each instrument and test.

### Materials and methods

Blood was collected from 10 apparently healthy Caucasian adults four times per week for six weeks. Serum specimens were stored at −20°C and −70°C for subsequent batch analysis when serum from each phlebotomy was analysed twice on both instruments. For the last two weeks the volunteers ingested 1 g of ascorbic acid three times a day as part of a protocol to determine the effects of large doses of ascorbic acid on laboratory tests [1].

The following 19 tests were carried out on the SMAC on the 25th and 26th days after specimen collection was completed, using one aliquot stored at −70°C and one stored at −20°C: alanine aminotransferase (EC 2.6.1.2), albumin, alkaline phosphatase (EC 3.1.3.1), aspartate aminotransferase (EC 2.6.1.1), total bilirubin, calcium, carbon dioxide, chloride, cholesterol, creatine kinase (EC 2.7.3.2), creatinine, lactate dehydrogenase (EC 1.1.1.27), inorganic phosphorus, potassium, total protein, sodium, triglycerides, urea nitrogen and uric acid.

Serum stored at −20°C was assayed in duplicate on the Perkin-Elmer KA 150 enzyme analyser for alanine and aspartate aminotransferase, alkaline phosphatase, creatine kinase, and lactate dehydrogenase 34 to 38 days after the last specimen was collected. Detailed information about the volunteers, specimen collection and handling, analytical methods and data editing has been given by van Steirteghem [2].

The frequencies of the different error flags were tabulated for each assay using all specimens from the entire eight-week period. The means of the unflagged results for each test for each subject were calculated, and then the arithmetic deviation of each result from the mean of the subject's unflagged results was determined. The means and variances of these deviations were computed separately for the flagged and unflagged results. The means and variances of the two groups of deviations for each assay were compared using one-way analysis of variance and the F test [3]. For each assay the number of results more than three standard deviations from the mean of the subject's unflagged results were tabulated for both flagged and unflagged values.

### Results

The percentage of error flags for SMAC and KA 150 analyses are tabulated in tables 1 and 2. For SMAC tests the percentage of flagged results ranged from 0·4 (urea and creatine kinase) to 10 (bilirubin); and for the KA 150 enzyme analyser from 0·2 (alkaline phosphatase) to 11 (aspartate aminotransferase). The data were examined for the effect of vitamin C administration and storage temperature on the occurrence of error flags. For the SMAC, specimens collected during vitamin C administration had more error flags, regardless of storage temperature. Specimens stored at −70°C had more error flags regardless of whether vitamin C was being given. For the KA 150 analyses, similar effects attributed to vitamin C were not found. The relation of the flagged and unflagged results is shown in table 3. For 14 out of 19 SMAC tests and two out of five KA 150 tests, the flagged results were significantly higher or lower than the

*Dr van Steirteghem now works at the RIA Laboratorium, Academisch Ziekenhuis, Vrije Universiteit Brussel, Laarbeeklaan 101, B 1090 Brussels, Belgium; and Dr Young at the Department of Laboratory Medicine, Mayo Clinic, Rochester, Minnesota 55901, USA.*

 0142-0453/82/0402-0072 $05.00 © 1982 Taylor & Francis Ltd

*Table 1. Percentage of flagged or absent SMAC results for 487 specimens.*

| | Flagged results (%) | Absent* results (%) |
|---|---|---|
| Alanine aminotransferase | 3·0 | 22·0 |
| Albumin | 1·0 | |
| Alkaline phosphatase | 1·0 | 4·0 |
| Asparatate aminotransferase | 2·0 | 1·0 |
| Bilirubin (total) | 10·0 | 2·0 |
| Calcium | 3·0 | |
| Carbon dioxide | 4·0 | 2·0 |
| Chloride | 6·0 | |
| Cholesterol | 6·0 | 1·0 |
| Creatine kinase | 0·4 | |
| Creatinine | 1·0 | |
| Lactate dehydrogenase | 3·0 | |
| Phosphorus | 1·0 | |
| Potassium | 2·0 | |
| Protein (total) | 5·0 | |
| Sodium | 2·0 | |
| Triglycerides | 1·0 | 4·0 |
| Urea nitrogen | 0·4 | |
| Uric acid | 1·0 | 0·4 |

**SMAC did not report any result because it detected unacceptable analytical conditions.*

unflagged values ($p<0{\cdot}01$ in each case). Furthermore, the variance of the deviations of the flagged results was significantly greater for 17 SMAC tests and three KA 150 tests ($p<0{\cdot}01$ in each case).

Results deviating from the expected value by more than three standard deviations were more frequent for flagged than unflagged results for 17 SMAC tests and all five KA 150 tests (table 4). The histograms in figure 1 show the excessive scatter of flagged results.

*Table 2. Percentage of flagged KA 150 results for 492 specimens.*

| | Error flag | | |
|---|---|---|---|
| | AC[1] % | DC[2] % | HI[3] % |
| Alanine aminotransferase | 0·4 | 1·0 | 5·0 |
| Alkaline phosphatase | — | 0·2 | — |
| Asparate aminotransferase | 0·2 | 2·0 | 9·0 |
| Creatine kinase | 2·0 | | 0·4 |
| Lactate dehydrogenase | 0·4 | 0·4 | 6·0 |

1. *AC signifies a nonlinear curve with upward concavity.*
2. *DC signifies a nonlinear curve with downward concavity.*
3. *HI signifies excessive absorbance.*

*All results with an error of HI were from the hyperlipaemic subject.*

*Table 3. Comparison of within-subject means and variances of flagged and unflagged results.*

| *KA 150* | Differences in means[1] | *p* | Ratio of variances[2] | *p* |
|---|---|---|---|---|
| Alanine aminotransferase | 7 U/L | <0·0001 | 120 | <0·001 |
| Alkaline phosphatase | −21 U/L | NS[3] | | |
| Aspartate aminotransferase | 2 U/L | <0·004 | 2 | <0·001 |
| Creatine kinase | 8 U/L | NS | 0·4 | NS |
| Lactate dehydrogenase | −2 U/L | NS | 2 | <0·005 |
| SMAC | | | | |
| Alanine aminotransferase | −2 U/L | NS | 8 | <0·001 |
| Albumin | −13 g/l | <0·0001 | 44 | <0·001 |
| Alkaline phosphatase | 3 U/l | NS | 38 | <0·001 |
| Aspartate aminotransferase | −4 U/l | NS | 8 | <0·001 |
| Bilirubin (total) | 0·8 mg/l (1·3 μmol/l) | <0·002 | 4 | <0·001 |
| Calcium | −7 mmol/l | <0·0001 | 45 | <0·001 |
| Carbon dioxide | −4 mmol/l | <0·0001 | 6 | <0·001 |
| Chloride | −28 mmol/l | <0·0001 | 45 | <0·001 |
| Cholesterol | −240 mg/l (−0·62 mmol/l) | <0·0001 | 16 | <0·001 |
| Creatine kinase | −23 U/l | NS | 0·3 | NS |
| Creatinine | −5·8 mg/l (−51 μmol/l | <0·0001 | 3 | <0·001 |
| Lactate dehydrogenase | −26 U/l | <0·0001 | 7 | <0·001 |
| Phosphorus | −24 mg/l (0·78 mmol/l) | <0·0001 | 12 | NS |
| Potassium | −2 mmol/l | <0·0001 | 22 | <0·001 |
| Protein (total) | −9·2 g/l | <0·0001 | 34 | <0·001 |
| Sodium | −37 mmol/l | <0·0001 | 22 | <0·001 |
| Triglycerides | −780 mg/l (−0·86 mmol/l) | <0·0001 | 8 | <0·001 |
| Urea nitrogen | −38 mg/l (−1·4 mmol/l) | NS | 14 | <0·001 |
| Uric acid | −8 mg/l (−46 μmol/l) | <0·003 | 15 | <0·001 |

1. *Mean of flagged results minus mean of unflagged results.*
2. *Variance of flagged results divided by variance of unflagged results.*
3. *NS signifies p>0·01.*

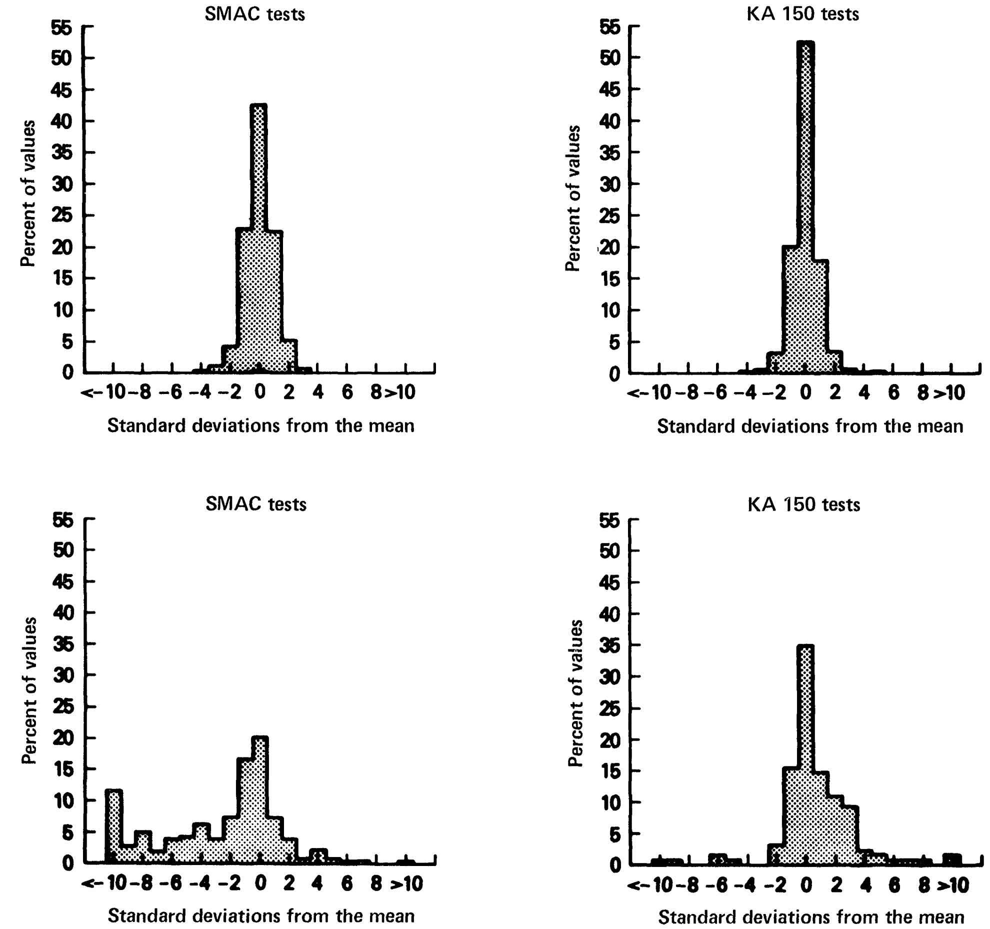

*Figure 1. Comparison of distributions of results without error flags (top) and with error flags (bottom). Results have been expressed as the number of standard deviations by which they differ from the mean of unflagged results for the subject and test involved.*

*Table 4. Percent of flagged and unflagged results more than three standard deviations from the mean of unflagged results.*

| *KA 150* | Percentage deviant values[1] Unflagged | Flagged |
|---|---|---|
| Alanine aminotransferase | 1·0 | 25 |
| Alkaline phosphatase | 2·0 | 100 |
| Aspartate aminotransferase | 1·0 | 9 |
| Creatine kinase | 3·0 | 36 |
| Lactate dehydrogenase | 3·0 | 62 |
| *SMAC* | | |
| Alanine aminotransferase | 2·0 | 15 |
| Albumin | 1·0 | 100 |
| Alkaline phosphatase | 1·0 | 40 |
| Asparate aminotransferase | 1·0 | 70 |
| Bilirubin (total) | 0·5 | 18 |
| Calcium | 2·0 | 71 |
| Carbon dioxide | 0·0 | 27 |
| Chloride | 3·0 | 90 |
| Cholesterol | 0·4 | 18 |
| Creatine kinase | 2·0 | 0 |
| Creatinine | 0·0 | 100 |
| Lactate dehydrogenase | 1·0 | 31 |
| Phosphorus | 1·0 | 100 |
| Potassium | 1·0 | 100 |
| Protein (total) | 2·0 | 27 |
| Sodium | 2·0 | 100 |
| Triglycerides | 1·0 | 0 |
| Urea nitrogen | 0·4 | 50 |
| Uric acid | 0·4 | 33 |

1. *Percentage of results more than three standard deviations from the mean of unflagged results.*

### Discussion

One subject was found to have a hyperlipidaemia. His specimens accounted for over 50% of the error codes for KA 150 alanine aminotransferase, asparate aminotransferase, creatine kinase, and lactate dehydrogenase analyses and SMAC lactate dehydrogenase and total protein analyses.

In evaluating the performance of algorithms for printing error flags, real human specimens, rather than artificial control sera, should be used. Control materials may not behave in the same way as patient specimens because of the presence of stabilizers, preservatives, non-human enzymes, turbidity, altered protein composition and so on.

The analyses indicate that for both the SMAC and KA 150 enzyme analyser, results with error flags tend to have a systematic bias, a larger variability and a higher percentage of grossly deviant results. We conclude that SMAC and KA 150 error flags indicate results which are likely to be wrong and therefore require repeat analysis.

### References

1. Van Steirteghem, A. C., Robertson, E. A. and Young, D. S., *Clinical Chemistry*, **24** (1978), 54.
2. Van Steirteghem, A. C., Robertson, E. A. and Young, D. S., *Clinical Chemistry*, **24** (1978), 212.
3. Dixon, W. J., *Biomedical Computer Programs P-Series* (University of California Press, Berkeley, 1977), p. 185.

